# Intraosseous Synovial Sarcoma of the Proximal Tibia

**DOI:** 10.1155/2011/184891

**Published:** 2011-05-14

**Authors:** Sarah E. Beck, G. Petur Nielsen, Kevin A. Raskin, Joseph H. Schwab

**Affiliations:** ^1^Department of Orthopaedics, Massachusetts General Hospital and Harvard Medical School, Boston, MA 02114, USA; ^2^Department of Pathology, Massachusetts General Hospital and Harvard Medical School, Boston, MA 02114, USA

## Abstract

Synovial Sarcoma is a malignant mesenchymal tumor that comprises 5–10% of all soft tissue sarcomas. The mean age of onset is thirty years old. Intraosseous presentation is very rare and has only been documented a few times. We report herein a case of a 53-year-old man with synovial sarcoma arising in the left proximal tibia. The patient underwent a wide surgical resection and reconstruction, followed by adjuvant chemotherapy. Three years later, the patient developed a local recurrence that resulted in an above-the-knee amputation. Eight months later, the patient has completed chemotherapy and is without signs of recurrence. The current recommended treatment for synovial sarcoma is wide surgical resection followed by chemotherapy as well as long-term followup. Despite improved surgical techniques, long-term survival rates remain low.

## 1. Introduction

Synovial sarcoma usually arises in the soft tissues of the extremities and rarely presents as a primary bone tumor. The first reported case of an intraosseous synovial sarcoma occurred in the proximal tibia [1]. Since then, only two cases have been completely documented with histological and molecular genetic confirmation [2, 3]. Five additional incompletely documented cases have been reported [4–8].

Synovial sarcoma is a malignant soft tissue tumor that comprises 5–10% of all soft tissue sarcomas [3]. It most often affects adolescents and young adults and has a mean age of onset of 30 years [9], with less than 10% of patients being over the age of 60 [10]. 

It usually arises in the lower extremities [4]. The large majority of tumors appear near large joints, but it has also been reported to arise in a variety of other locations such as in the head and neck [11], chest [12], and abdomen [13]. While 20% show erosion or invasion of adjacent bone [14], primary synovial sarcoma of bone is extremely rare [3]. We report a case of primary intraosseous synovial sarcoma arising in the proximal tibia.

## 2. Case Report

**Figure 1:**
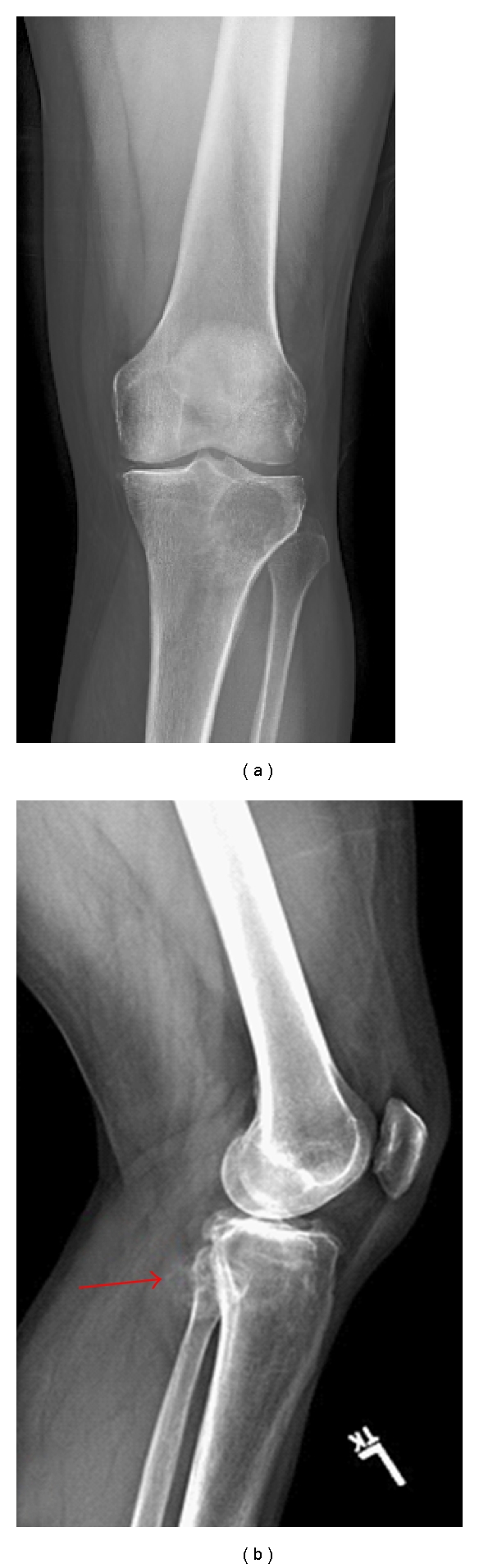
(a) A/p radiograph of the left knee shows a round lucency at the lateral tibial plateau with well-defined proximal margins and a less well-defined distal margin.(b) Lateral radiograph shows a stippled mineralization (arrow) projecting into the soft tissues posteriorly, measuring approximately 2.5 cm in maximal diameter.

A 53-year-old man presented with a 3-month history of left knee pain. He had difficulty weight bearing and complained of swelling of the calf as well as a lack of sensation in the toes. X-rays showed a large, 5 cm round lucency involving the lateral proximal portion of the tibia (Figure 1(a)). The lateral view showed stippled mineralization extending into the soft tissues posteriorly (Figure 1(b)). MRI showed a large mass in the tibial plateau of the left proximal tibia with a soft tissue extension postero-laterally. 

**Figure 2:**
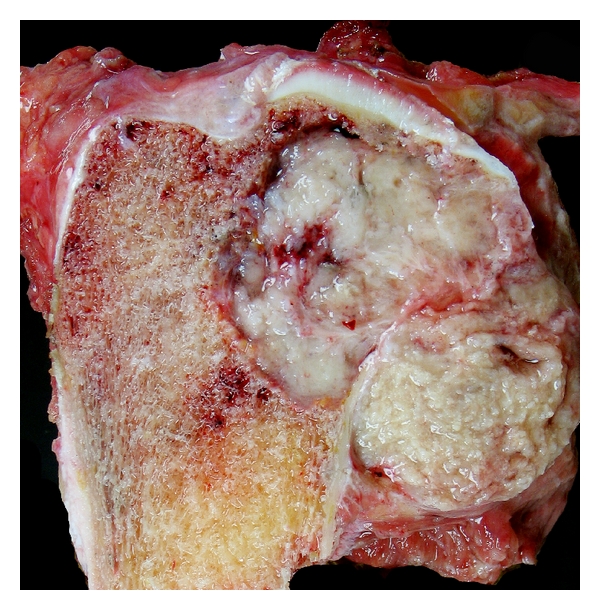
Grossly the tumor is eccentrically located in the proximal tibia. It has a tan-grey fleshy cut surface, which breaks through the cortex and extends into adjacent soft tissue (right).

**Figure 3:**
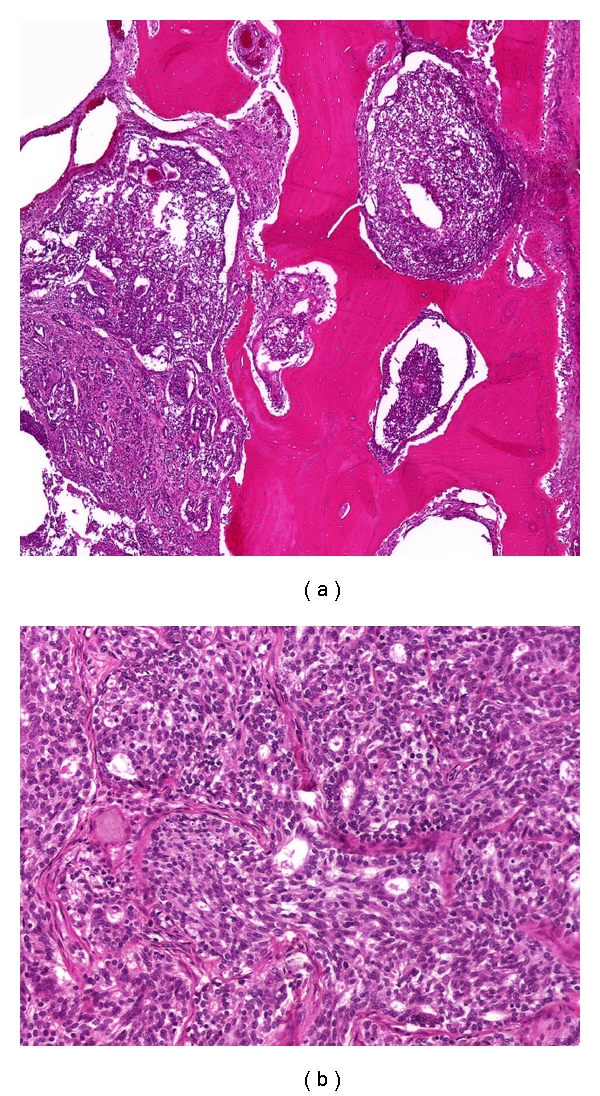
(a) Photomicrograph of intraosseous synovial sarcoma. The tumor is present in the medullary cavity of the bone (left), infiltrates the cortex, and expands the haversian systems. (Stain, hematoxylin, and eosin; original magnification, ×40). (b) Photomicrograph of intraosseous synovial sarcoma. The tumor shows a typical biphasic pattern with glandular differentiation admixed with spindle-shaped cells. (Stain, hematoxylin, and eosin; original magnification, ×400).

**Figure 4:**
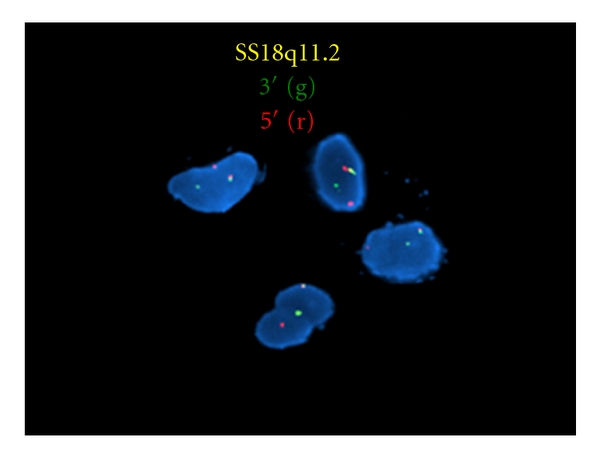
Interphase nuclei showing an abnormal hybridization pattern using the Vysis LSI SS18 Dual Color, Break Apart Rearrangement Probe at 18q11.2 (Abbott Molecular). SS18 gene rearrangement appeared as a separation of the red-orange and green signals from the normal fusion signal (yellow).

**Figure 5:**
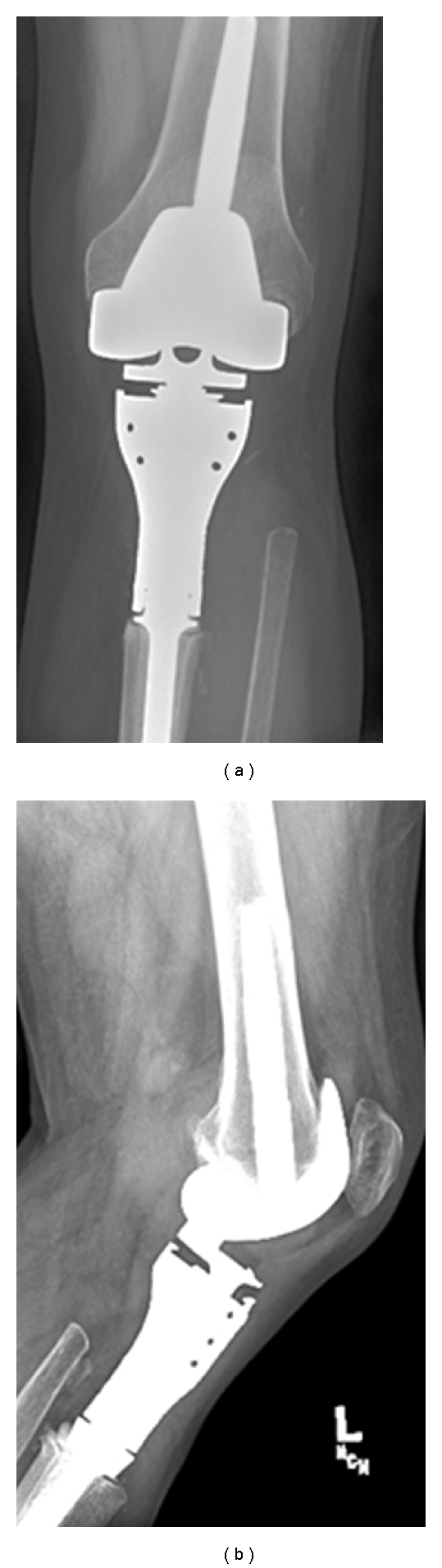
Postoperative anterior posterior, (a) and lateral (b) radiographs of the left knee demonstrate the endoprosthetic reconstruction of the proximal tibia and knee joint.

Chest X-ray was negative for metastatic disease. A CT scan of the chest, pelvis, and abdomen along with a bone scan were negative except for an increased uptake in the proximal tibia. A core needle biopsy was performed revealing a high-grade sarcoma. A proximal tibial resection with negative margins was performed. Grossly, the tumor was tan-yellow, arising in the proximal tibia and extending into the adjacent soft tissue. The intraosseous component measured 6.0 × 5.0 × 3.5 cm and the extraosseous soft tissue component measured 4.0 × 3.0 × 2.0 cm (Figure 2). Histologically, the tumor showed the characteristic features of a biphasic synovial sarcoma, containing obvious glandular differentiation admixed with a malignant spindle cell component (Figures 3(a) and 3(b)). Immunohistochemical studies showed that the neoplastic cells were positive for keratin, cytokeratin 7, and epithelial membrane antigen (EMA). Fluorescence in situ hybridization (FISH) analysis was performed on interphase nuclei isolated from f paraffin-embedded 50 *μ*m tissue sections, for the detection of possible t(X;18)(p11;q11). An SS18 rearrangement was observed in 49/50 nuclei examined, confirming the diagnosis of synovial sarcoma (Figure 4). An endoprosthetic reconstruction was performed using a cemented prosthesis (Figures 5(a) and 5(b)). The patient was treated with adjuvant chemotherapy including dacarbazine, doxorubicin, ifosfamide, and mesna. 

**Figure 6:**
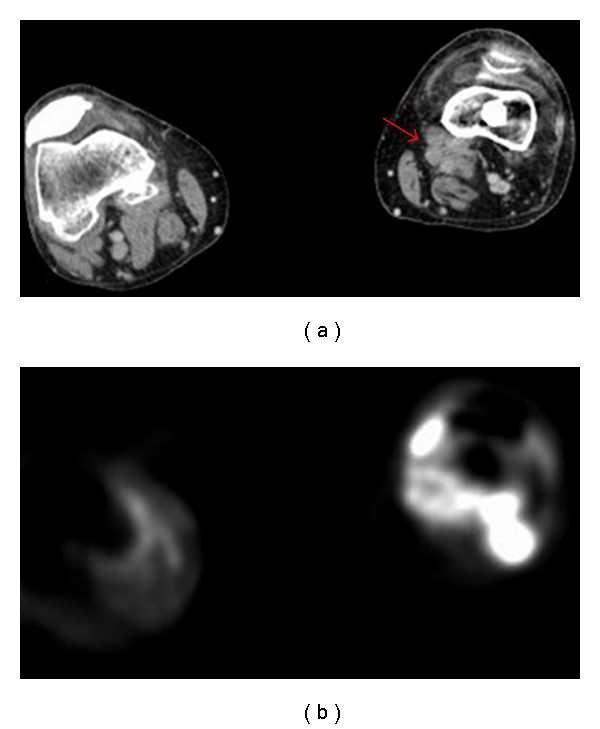
(a) A preoperative CT scan reveals multiple soft tissue nodules (arrow) above and below the knee including areas medial, lateral, anterior, posterior and in the suprapatellar recess. (b) A preoperative PET scan shows intense FDG uptake in the multiple soft tissue nodules above and below the knee consistent with recurrent disease.

Three years later, the patient presented again with left knee pain. A PET/CT scan revealed multiple soft tissue nodules above and below the knee (Figures 6(a) and 6(b)). A CT scan of the chest was negative. A fine needle aspiration revealed recurrent synovial sarcoma. The patient underwent an above-the-knee amputation with prosthetic reconstruction.

The resection specimen showed soft tissue nodules above and below the knee with a large soft tissue component as well as involvement of the distal femur. The tumor measured 4 × 3 × 2.5 cm in its largest dimension. Again, the tumor was noted to be biphasic synovial sarcoma. The patient was restarted on adjuvant chemotherapy including mesna, adriamycin, ifosfamide, and dacarbazine. Eight months have passed since the amputation. He continues adjuvant chemotherapy and has no other sites of disease.

## 3. Discussion

Synovial sarcoma was originally named in 1936 by Knox as it was believed to be of synovial cell origin [3]. This term is misleading as many cases of synovial sarcoma have been found to originate elsewhere where no synovial tissue is present, and at this time, the tumor is regarded as a neoplasm of “uncertain histogenesis” [15]. Many cases occur outside of the synovium and less than 5% of synovial sarcomas actually originate in a joint or bursa [14]. While 20% of synovial sarcomas show invasion of local bone, it is extremely rare for it to present as a primary bone tumor [14]. 

We believe that our case represents a primary bone tumor because the osseous extent of the tumor is greater than the soft tissue component. In addition, our imaging studies suggest that the epicenter of the tumor began in bone and grew secondarily into soft tissues.

Radiographically, synovial sarcoma presents with MR findings of heterogeneous intermediate signal intensity on T1W1, heterogeneous high signal intensity on fat-suppressed T2W1, and heterogeneous contrast enhancement [3]. Histologically, synovial sarcoma is classified as biphasic, monophasic, or poorly differentiated [15], with the biphasic being the most common. Synovial sarcoma is locally aggressive, and distant metastases are common [9]. Additionally the diagnosis of synovial sarcoma can be confirmed genetically with the detection of t(X;18)(p11.2;q11.2). This translocation involves the SS18 (a.k.a.SYT) gene on chromosome 18 and either the *SSX1*, *SSX2*, or rarely the *SSX4* gene on the X chromosome [15]. This translocation occurs in more than 90% of all synovial sarcomas [10]. 

Synovial sarcoma of bone was first reported in 1997 [1]. Since then confirmed cases of primary synovial sarcoma of bone have been found in the sacrum [6], distal tibia [7], mandible [8], elbow [2, 4], distal tibia [3], and sternum [5]. Due to its aggressive nature, recommended treatment is a combination of wide surgical resection to achieve clear margins, followed by chemotherapy and/or irradiation. Long-term follow-up is necessary due to this tumor's high rate of recurrence and metastasis [16].

Development of distant metastasis and decreased disease-specific survival has been correlated to a large tumor size (greater than 5 cm) and invasion of bone, nerve, or vascular structures [16, 17]. In 1950, a study of 60 patients treated only with simple excision showed a 63% local recurrence rate [18]. As a result of this study, simple excision as the solitary form of treatment is no longer being practiced. 

Studies have shown clear indications for the use of irradiation therapy for local control of synovial sarcoma. In 2000, Lewis et al. showed a local recurrence of only 10% in their series of 112 patients with primary localized tumors of the extremity [17]. Approximately half of the patients in this study also underwent adjuvant irradiation. The authors thus attributed this better local recurrence rate, in comparison to the 1950 study, to improved surgical techniques as well as to the use of adjuvant radiation [17].

While the rate of local recurrence has diminished, the rate of distant metastasis is still a challenge for clinicians. Lewis et al. found a 40% chance of metastasis within five years, despite wide surgical resection with negative margins [17]. Five-year survival rate has been reported to be as low as 25–50%, while ten-year survival rate is only 10–15% [8]. While the use of chemotherapy for systemic control has been previously questioned, a recent study has shown that chemotherapy treatment is significantly associated with an increased 4-year disease-specific survival (DSS) [16]. The 4-year DSS was 88% in the ifosfamide- treated patients and 67% in the patients that did not receive chemotherapy [16].

We chose chemotherapy treatment over radiation following surgical resection in order to decrease the chance of distant metastasis. The primary tumor, being larger than 5 cm, had an increased chance of distant metastasis [17]. There was also a significant soft tissue component. The recurrent tumor displayed multiple soft tissue nodules that indicated it was at least a regional, if not a systemic problem that would also increase the chance for metastasis. 

Overall, these recent studies are showing a positive progression of treatment methods. The lower rates of local recurrence are promising, but the low five-year survival rates and the high chance of distant metastasis still show the need for the development of adequate systemic therapy. Due to the aggressive nature of this disease and the high rate of metastasis, long-term follow-up is essential.
